# Refractive outcomes and optical quality after implantation of posterior chamber phakic implantable collamer lens with a central hole (ICL V4c)

**DOI:** 10.1186/s12886-018-0805-3

**Published:** 2018-06-14

**Authors:** Huamao Miao, Xun Chen, Mi Tian, Yingjun Chen, Xiaoying Wang, Xingtao Zhou

**Affiliations:** Department of Ophthalmology, Eye and ENT Hospital of Fudan Universtity, NHC Key Laboratory of Myopia (Fudan Universtity), No.83 FenYang Road, Shanghai, 200031 People’s Republic of China

**Keywords:** Myopia, Phakic intraocular Lens, V4c, Refractive outcomes, Optical quality, Intraocular scattering, Intraocular pressure

## Abstract

**Background:**

To investigate refractive outcomes and optical quality after implantation of posterior chamber phakic implantable collamer lens with a central hole (ICL V4c) to correct high myopia.

**Methods:**

Sixty seven eyes of 38 patients who underwent ICL V4c implantation were enrolled. The mean preoperative spherical equivalent (SE) was − 12.44 ± 3.15 D (range: − 6.63 to − 20.50 D). The refractive outcomes and optical quality of the eyes at postoperative 1 and 3 months were evaluated and compared.

**Results:**

At 3 months postoperatively, the mean safety and efficacy indexes were 1.33 ± 0.22 and 1.14 ± 0.23, respectively. The mean SE was − 0.32 ± 0.52 D; no patient lost 1 or more lines of corrected distance visual acuity (CDVA), 13% remained unchanged, 45% gained 1 line and 42% gained 2 or more lines. The mean modulation transfer function cutoff frequency (MTF_cutoff_), Strehl in two dimensions ratio, and objective scatter index (OSI) were 38.20 ± 9.96 cycles per degree, 0.21 ± 0.06, and 1.00 ± 0.73, respectively. No significant difference was found in any of the above parameters (*P* > 0.05) between 1 and 3 months. The postoperative intraocular pressure (IOP) did not change when compared with preoperative values (*P* > 0.05).

**Conclusions:**

ICL V4c implantation is a safe, effective, and stable solution for high myopia. Patients will acquire high and stable postoperative optical quality. ICL V4c implantation has little influence on IOP.

## Background

The prevalence of myopia in China is very high. Most patients seeking refractive surgery treatments suffer from moderate to high myopia. Laser corneal refractive surgeries to correct myopia have gained wide acceptance in China. However, the surgery is not safe for patients with high level of refractive error and relatively thin corneas, as these conditions could increase the risk of postoperative corneal ectasia [1]. Phakic intraocular lens implantation could correct a wider range of refractive errors and avoid decreases in corneal thickness; therefore, it would be a good option for patients with these conditions. Even in eyes with keratoconus, the posterior chamber toric phakic ICL was found to be effective in correcting high myopic astigmatism [2].

The posterior chamber phakic implantable collamer lens with a central hole (ICL V4c) is a newly developed kind of ICL, which is designed with a 360 μm central hole in the central optical zone. The advantage of the ICL V4c implantation is that it is less traumatic than the conventional ICL (without a central hole) implantation. Experimental and clinical studies conducted in non-Chinese countries have proved that, by improving the circulation of aqueous humour to the anterior surface of the crystalline lens, the specially designed central hole helps to maintain the intraocular pressure (IOP) without additional peripheral iridotomy [3, 4]. However, the peripheral iridotomy is an inevitable procedure in conducting conventional ICL (without a central hole) implantation. In addition, secondary anterior segment cataract formation is the main concern after implantation of a conventional ICL [5, 6]. According to the previous experimental results, the ICL V4c also holds the potential to reduce the risk of cataract formation [7–9]. Thus, it is considered that the ICL V4c could be a promising alternative to the conventional ICL for myopia correction.

This prospective study reported the refractive outcomes and optical quality in the 3 months after ICL V4c implantation in a group of Chinese adults with high myopia.

## Methods

### Subjects

In this prospective non-randomized study, 67 eyes of 38 consecutive patients (9 unilateral and 29 bilateral patients), 10 of which were male and 28 of which were female, were examined. The mean age was 28.61 ± 6.10 years (range: 18 to 40 years). Preoperatively, all the patients underwent routine ophthalmic examinations at the Refractive Surgery Center of the Department of Ophthalmology, Eye and ENT Hospital of Fudan University (Shanghai, People’s Republic of China) and met the surgical requirements for ICL V4c (STAAR Surgical Company, Monrovia, California, USA) implantation. Inclusion criteria were: aged between 18 and 40 years, spherical refraction error between − 6.00 D and − 20.00 D, astigmatism of up to − 5.00 D, corrected distance visual acuity (CDVA) of 20/40 or better, anterior chamber depth of ≥2.8 mm, and endothelial cell density of ≥2000 cell/mm^2^. Exclusion criteria were: a history of certain ocular diseases (suspicion of keratectasia, cornea or lens opacity, retinal detachment, glaucoma, macular degeneration, or neuro-ophthalmic disease), a history of ocular surgery, inflammation or trauma, and systemic disease.

This study adhered to the Declaration of Helsinki and was approved by the Ethical Committee Review Board of Fudan University Eye and ENT Hospital. All patients gave written informed consent after the possible risks and benefits of the study were explained.

### ICL V4c

The ICL V4c is a plate-haptic single-piece intraocular lens made of collamer. It has a central convex–concave optical zone and incorporates a forward vault to minimize contact with the crystalline lens. A 360 μm central hole was included to improve aqueous humor circulation, which eliminates the need for preoperative laser peripheral iridotomy [7]. The ICL V4c corrects − 0.50 D to − 18.00 D myopic spherical refraction and up to − 5.00 D cylindrical refraction. There are 4 sizes: 12.1 mm, 12.6 mm, 13.2 mm, and 13.7 mm. Power calculation of the ICL V4c was performed by the manufacturer using a modified vertex formula, according to the provided preoperative refractive parameters. The size of the implanted ICL V4c was determined based on the white-to-white horizontal corneal diameter and anterior chamber depth. Toric ICL V4c is designed to correct both spherical and cylindrical diopters. In the present study, toric ICL V4c was implanted in 30 eyes, with a mean preoperative cylindrical diopter of − 2.64 ± 1.06D (range, − 1.25D to − 5.00D); the other 37 eyes chose the ordinary ICL V4c and the mean preoperative cylindrical diopter was − 0.91 ± 0.54D (range, − 0.25D to − 2.50D).

### Surgical technique

ICL V4c implantation procedures were performed by two experienced surgeons (XZ and XW). Pupils were dilated before surgery. After injection of 1% sodium hyaluronate into the anterior chamber via a puncture site at the 6 o’clock position of the cornea, an ICL V4c was implanted via a 3.0 mm temporal corneal incision using an injector cartridge and then was placed in the posterior chamber. After that, the viscoelastic surgical agent was completely removed using a balanced salt solution, and a miotic agent was instilled. Postoperative medications included antibiotics eye drops, non-steroidal anti-inflammatory eye drops, steroidal eye drops, and artificial eye drops. At 1 and 3 months postoperatively, the following parameters were collected: manifest refraction, uncorrected distance visual acuity (UDVA), CDVA, IOP measured with a non-contact tonometer (NCT; Canon, Japan), retinal image quality, and intraocular scattering. Four patients did not attend the 1-month follow-up. The safety index was calculated as the ratio of the CDVA at 3 months to the corresponding preoperative CDVA, and the efficacy index was the ratio of the UDVA at 3 months to the preoperative CDVA [10].

### Retinal image quality and intraocular scattering measurement

Retinal image quality and intraocular scattering were objectively measured using a double-pass optical quality analysis system (OQAS II; Visiometrics, Terrassa, Spain). Before the measurements, the cylindrical diopter of − 0.50D or higher should be corrected using an external lens and the spherical diopter was automatically corrected by the double-pass system. The system has been used in our clinical practice to evaluate optical quality in myopic patients and after corneal refractive surgeries, and the same methods were applied in the present study [10–12]. In brief, a two-dimension modulation transfer function (MTF) profile was calculated from the image of a light source (780 nm laser diode) reflected on the retina using the Fourier transform. Five representative indexes were derived from the MTF profile for retinal image quality evaluation, including MTF cutoff frequency (MTF_cutoff_), Strehl in 2 dimensions (Strehl2D) ratio, and OQAS values (OV) at 3 levels of contrast.

MTF_cutoff_ represents the spatial frequency at which the MTF value is 0.01. Theoretically, an MTF_cutoff_ of 30 cycles per degree (cpd) usually corresponds to 20/20 visual acuity, and the maximum value is no more than 60 cpd [13]. The Strehl2D ratio is a more comprehensive index for evaluating optical quality. It is the ratio between the aberrated eye and the ideal aberration-free eye in the MTF profiles, and the value ranges between 0 and 1.0. OVs of 100, 20, and 9% were calculated as the spatial frequencies at the MTF values of 0.01, 0.05, and 0.1, respectively, divided by 30 cpd [13]. These are normalized values that are comparable to the standard decimal measurement for visual acuity. Higher values indicate higher optical quality. The above parameters are independent of retinal and neural factors.

The double-pass system uses objective scatter index (OSI) as an objective parameter to estimate intraocular scattering. The OSI is calculated as the ratio of the amount of light in the peripheral zone (an annular area of 12 and 20 min) to the central zone (central peak of 1 min of arc) of the retinal image [13–15]. An OSI value of less than1.0 indicates low scattering.

### Statistical analysis

Refractive outcome graphs were plotted using Microsoft Excel according to the refractive outcomes at 3 months in all the 67 eyes of the 38 patients. All statistical analyses were performed using the software Statistical Package for the Social Sciences (SPSS) Version 20.0 (SPSS, Chicago, IL, USA). The Kolmogoro–Smirnov test was used to determine if a variable is normally distributed. If both eyes were recruited in the study, only one eye was selected at random for comparison analysis, at last, 35 eyes of the 35 patients whose data were available in both follow-ups were selected. According to each variable’s distribution, a paired *t* test or Wilcoxon signed-rank test was chosed to compare the parameters between time points (1 and 3 months postoperatively).

## Results

Preoperatively, the mean spherical equivalent (SE) of the 67 eyes was − 12.44 ± 3.15 D (range: − 6.63 to − 20.50 D), the mean sphere was − 11.60 ± 3.05 D (range: − 6.25 to − 19.25 D), the mean cylinder was − 1.69 ± 1.18 D (range: − 0.25 to − 5.00 D), and the mean logMAR CDVA was 0.04 ± 0.08 (range: − 0.08 to 0.30).

**Fig. 1:**
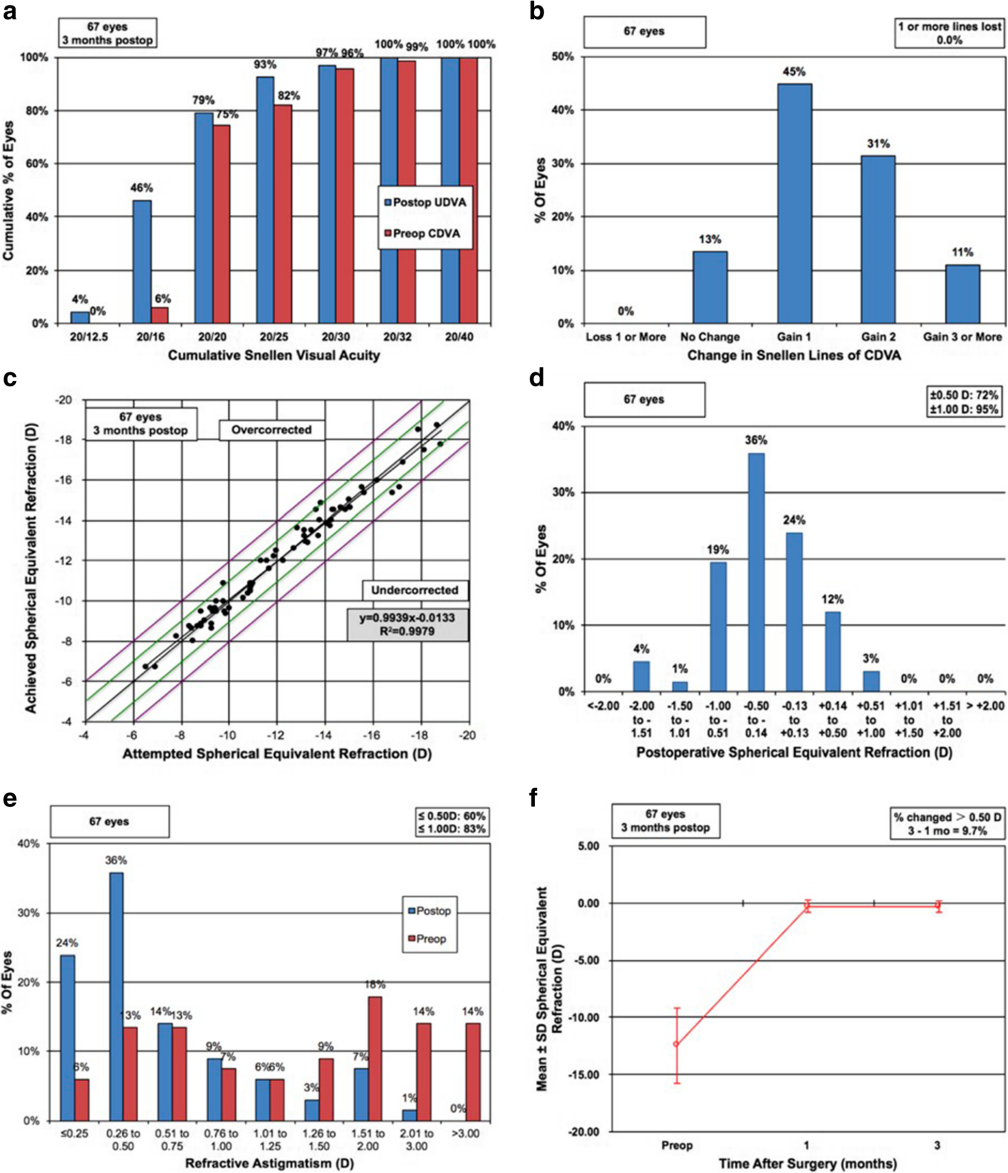
Refractive outcomes at 3 months postoperatively in 67 eyes with high myopia after implantation of posterior chamber phakic implantable collamer lens (ICL V4c) with a central hole, including (**a**) postoperative uncorrected distance visual acuity, (**b**) change in corrected distance visual acuity, (**c**) attempted versus achieved spherical equivalent, (**d**) spherical equivalent refractive accuracy, (**e**) refractive astigmatism, and (**f**) stability of spherical equivalent refraction. UDVA = uncorrected distance visual acuity; CDVA = corrected distance visual acuity; D = diopters; Postop = postoperative; Preop = preoperative; SD = standard deviation; mo = months

At 3 months postoperatively, the mean values of the safety and efficacy indexes of all 67 eyes were 1.33 ± 0.22 and 1.14 ± 0.23, respectively, and the mean logMAR CDVA and UDVA were − 0.08 ± 0.07 and − 0.01 ± 0.09, respectively. The refractive outcomes of all 67 eyes at 3 months postoperatively were summarized in Fig. 1. All the eyes had a CDVA or UDVA of 20/40 or better at 1 and 3 months (Fig. 1a). Eyes with a CDVA of 20/20 or better increased from preoperative 70 to 94% at 3 months postoperatively. None of the 67 eyes lost 1 or more lines of CDVA, 13% remained unchanged, 45% gained 1 line, and 42% gained 2 or more lines (Fig. 1b). A scatterplot and the best linear fit line (*r*^*2*^ = 0.9979) of the attempted versus the achieved SE correction are shown in Fig. 1c. Of the 67 eyes, 92.54% were within ±1.00 D (Fig. 1c, green lines) and 100% were within ±2.00 D of the desired SE refraction (Fig. 1c, purple lines). At 3 months, 72, 95, and 100% of the eyes had an SE refraction within ±0.5, ± 1.0, and ± 2.0 D, respectively (Fig. 1d), and 60, 83, and 99% had astigmatism within ±0.5, ± 1.0, and ± 2.0 D, respectively (Fig. 1e). The mean SE increased from preoperative − 12.44 ± 3.15 D to − 0.30 ± 0.52 D at 3 months, and the SE refraction barely changed from 1 to 3 months (Fig. 1f). When the refractive parameters were compared between 1 and 3 months, no significant differences were observed regarding refractive sphere, refractive cylinder, SE, logMAR UDVA or CDVA, safety index, or efficacy index (*P* > 0.05; Table 1).

**Table 1:** Refractive Outcomes at 1 and 3 Months after ICL V4c Implantation for Correcting High Myopia. (*n* = 35)

Parameters	1 Month		3 Months		*P*
	Mean	SD	Mean	SD	
Sphere (D)	0.09	0.56	0.10	0.54	0.906
Cylinder (D)	−0.73	0.54	−0.71	0.52	0.786
SE (D)	−0.27	0.51	−0.26	0.47	0.793
logMAR UDVA	−0.01	0.11	−0.01	0.09	0.580
logMAR CDVA	−0.06	0.09	−0.08	0.08	0.071
Efficacy index	1.11	0.22	1.13	0.24	0.797
Safety index	1.25	0.20	1.31	0.22	0.060

*ICL V4c* Posterior Chamber Phakic Implantable collamer lens With a Central Hole, *n* number of eyes, *SD* standard deviation, *SE* spherical equivalent refraction, *D* diopters, *logMAR* the logarithm of the minimal angle of resolution, *UDVA* uncorrected distance visual acuity, *CDVA* corrected distance visual acuity

At 3 months postoperatively, the mean MTF_cutoff_ was 38.20 ± 9.96 cpd (range: 13.14 to 55.59 cpd), the mean Strehl2D ratio was 0.21 ± 0.06 (range: 0.08 to 0.37), and the mean OSI was 1.00 ± 0.73 (range: 0.24 to 3.47) in all 67 eyes. The mean OVs at 100, 20, and 9% contrasts, were 1.27 ± 0.33 (range: 0.44 to 1.85), 1.27 ± 0.40 (range: 0.39 to 2.13), and 1.24 ± 0.43 (range: 0.37 to 2.34), respectively. No significant difference was found between 1 and 3 months postoperatively in any of the above optical quality-related parameters (*P* > 0.05; Table 2).

**Table 2:** Optical Quality at 1 and 3 Months after ICL V4c Implantation for Correcting High Myopia (*n* = 35)

Parameters	1 Month		3 Months		*P*
	Mean	SD	Mean	SD	
MTF_cutoff_ (cpd)	38.49	10.35	37.64	9.74	0.475
Strehl2D Ratio	0.22	0.07	0.21	0.06	0.587
OV100%	1.28	0.35	1.25	0.32	0.475
OV20%	1.30	0.43	1.25	0.39	0.408
OV9%	1.30	0.47	1.24	0.44	0.401
OSI	1.00	0.83	0.98	0.71	0.954

*MTF*_*cutoff*_ modulation transfer function cutoff frequency, *cpd* cycles per degree, *Strehl2D Ratio* Strehl in two dimensions ratio, *OV* optical quality analysis system value, *OSI* objective scatter index, *SD* standard deviation

**Fig. 2:**
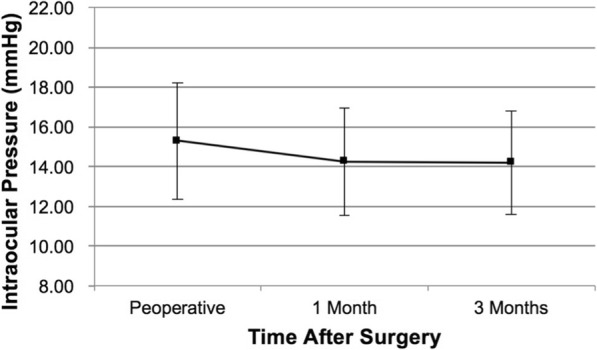
Intraocular pressure after ICL V4c implantation

The mean intraocular pressure of all the eyes was 15.30 ± 2.93 mmHg (range: 8.0 to 21.8 mmHg) preoperatively and 14.19 ± 2.62 mmHg (range: 9.3 to 20.5 mmHg) at 3 months postoperatively. No significant difference was observed between time points (*P >* 0.05; Fig. 2).

## Discussion

The newly developed ICL V4c has been reported in recent studies to produce encouraging clinical and experimental outcomes [3, 4, 16–27]. However, little is known about ICL V4c implantation in Chinese patients. Our study presented the visual outcomes, retinal image quality, intraocular scattering, and IOP results in 3 months after ICL V4c implantation in patients with high myopia.

In our study, none of the subjects lost their preoperative CDVA. Additionally, 87% of eyes gained 1 or more lines of CDVA, and the mean safety and efficacy indexes were over 1.1. In previous studies regarding visual outcomes 3 to 12 months after ICL V4c implantation, the mean values of the two indexes were over 1.0, and approximately 20 to 40% eyes gained 1 or more lines of CDVA [16–20]. The significant improvement in postoperative visual acuity in our study supports the safety and efficacy of ICL V4c implantation for myopia correction. Patients enrolled in the previous studies had spherical refraction ranging from − 2.75 to − 17.50 D, astigmatism of − 3.00 D or less, and a mean SE ranging from − 7.36 to − 9.32 D [16–20], while the values were relatively higher in our study, with spherical refraction ranging from − 6.25 to − 19.25 D, a maximum astigmatism of − 5.00 D, and a mean SE of − 12.44 D. In addition, part of the patients with astigmatism in the current study did not choose the toric ICL V4c. Even so, the results showed relatively satisfying predictability, with 95% or 83% of the eyes had SE or astigmatism within ±1.00 D at 3 months. Additionally, the refractive parameters, including visual acuity and manifest refraction, showed identical results at 1 and 3 months postoperatively, suggesting that patients’ refractive status could be stabilized soon after ICL V4c implantation.

Particular attention was paid to optical quality after refractive surgeries. Both the conventional ICL and the ICL V4c implantation procedures provide good optical and visual quality [16, 19, 24, 26–28]. Tian et al. found that the two kinds of ICL had similar efficacy of visual quality for high myopia, and also similar results in low-order aberrations, while high-order aberrations and spherical aberrations were higher after ICL V4c implantation [28]. In Kamiya’s study, the two ICLs produced similar optical quality and intraocular scattering results [24]. Kamiya et al. found that the mean OSI was 0.90 in 201 myopic adults with SE values ranging from − 1.25 to − 8.25 D, and that contrast sensitivity increased when OSI decreased [29]. Our previous study found that the mean MTF_cutoff_ was 35.15 cpd and the mean OSI was 0.74 in 274 myopic adults with SE values ranging from − 0.63 to − 14.25 D [11]. In the current study, the mean MTF_cutoff_ was 38.20 cpd and the mean OSI was 1.00 at 3 months, indicating high optical quality after ICL V4c implantation. Kamiya et al. and Huseynova et al. investigated optical quality at 3 months after ICL V4c implantation. The mean postoperative MTF_cutoff_, Strehl2D ratio, and OSI were 26.21 cpd, 0.16, and 1.16, respectively, in Kamiya et al.’s study [24], and the postoperative mean OSI was 1.08 in Huseynova et al.’s study [19]. When compared with the above two studies, retinal image quality and intraocular scattering were approximately higher and lower, respectively, in our study. This difference might be due to regional and ethnic differences, however, the reason needs to be explored further.

The identical optical quality results at 1 and 3 months suggested that the optical system could achieve stability shortly after ICL V4c implantation. In contrast, optical quality sometimes decreases after PRK and LASIK surgeries [30]. In our previous studies on optical quality after small incision lenticule extraction (SMILE) to correct moderate to high myopia correction, a temporary increase in intraocular scattering in the early stage (3 months postoperatively) was observed before the optical quality stabilized [10, 12]. The pIOL implantation procedures do not interfere with the central cornea and maintain the prolate shape of the cornea; thus, it should induce less high order aberrations than the corneal refractive surgeries and avoid the corneal wound healing process of the central optical zone in myopia correction [31–36]. These factors could contribute to the high stability and level of optical quality after ICL V4c implantation.

The stable IOP indicates that the central hole in ICL V4c could help maintain the IOP without peripheral iridotomy, which further certified the safety of ICL V4c implantation. As of yet, no study has reported secondary cataract after ICL V4c implantation [3, 4, 16–20]. ICL V4c improves the aqueous humor circulation to the anterior surface of the crystalline lens, thus reducing the risk of cataract formation [7–9]. Alfonso et al. reported that, for conventional ICLs, the mean time between phakic intraocular lens implantation and cataract surgery was 4.2 ± 1.8 years [5]. Therefore, longer-term observations of the above-mentioned parameters are needed for a more comprehensive understanding of ICL V4c implantation. In the present study, ocular scattering was measured with a double-pass system, which was suggested to be a useful tool in the preoperative evaluation of patients with early cataract [37]. Thus, OSI might be used as an objective parameter to monitor early cataract formation after ICL V4c implantation in further studies.

## Conclusions

In this 3-month prospective study, ICL V4c implantation was shown to be a safe, effective and stable way to correct high myopia. ICL V4c implantation has little influence on IOP. Patients could achieve high, stable postoperative optical quality shortly after the surgery.

## Abbreviations

CDVA: Corrected distance visual acuity
cpd: Cycles per degree
D: Diopters
ICL V4c: Posterior chamber phakic implantable collamer lens with a central hole
IOP: Intraocular pressure
logMAR: Logarithm of the minimal angle of resolution
MTF_cutoff_: Modulation transfer function cutoff frequency
N: Number of eyes
NCT: Non-contact tonometer
OQAS II: Double-pass optical quality analysis system
OSI: Objective scatter index
OV: Optical quality analysis system value
SD: Standard deviation
SE: Spherical equivalent
SPSS: Statistical package for the social sciences
Strehl2D Ratio: Strehl in 2 dimensions ratio
UDVA: Uncorrected distance visual acuity
